# Assessment of medical students’ Surgery knowledge based on Progress Test

**DOI:** 10.1590/0100-6991e-20233636-en

**Published:** 2023-11-18

**Authors:** Pedro Tadao Hamamoto, Angélica Maria Bicudo, Gerson Alves Pereira-Júnior

**Affiliations:** 1 - UNESP - Universidadde Estadual Paulista, Faculdade de Medicina de Botucatu - Botucatu - SP - Brasil; 2 - UNICAMP - Universidade Estadual de Campinas, Faculdade de Ciências Médicas - Campinas - SP - Brasil; 3 - FOB/USP - Curso de Medicina de Bauru - Bauru - SP - Brasil

**Keywords:** Surgery, Educational Measurement, Medical Education, Psychometrics, Cirurgia, Avaliação Educacional, Educação Médica, Psicometria

## Abstract

Progress Testing (PT) is an assessment tool whose use has grown throughout Brazil in the last decade. PT makes it possible to assess the students’ knowledge gain throughout the undergraduate course and, for their interpretations to be valid, their items (questions) must have adequate quality from the point of view of content validity and reliability of results. In this study, we analyzed the psychometric characteristics of the items and the performance of students in the content area of surgery from 2017 to 2023. For the analyses, we used the assumptions of Classical Test Theory, Bloom’s taxonomy and Cronbach’s alpha reliability coefficient. The items were easy (average difficulty index between 0.3-0.4), with fair to good discrimination (discrimination index between 0.3-0.4) and with a predominance of medium to high taxonomy. Reliability remained substantial over the years (>0.6). Students’ knowledge gain in surgery was found to be progressive and more important from the 3rd year of the undergraduate course, reaching approximately 70-75% in the 6th year. This measurements framework can be replicated in other contexts for a better understanding of student learning and for qualification of evaluation processes.

## RESUMO

O Teste de Progresso (TP) é uma ferramenta de avaliação cujo uso tem crescido em todo o Brasil na última década. O TP permite avaliar o ganho de conhecimento dos estudantes ao longo do curso de graduação e, para que suas interpretações sejam válidas, é preciso que seus itens (questões) tenham qualidade adequada do ponto de vista de validade de conteúdo e confiabilidade de resultados. Neste estudo, analisamos as características psicométricas dos itens e o desempenho dos estudantes na área de cirurgia do TP de 2017 a 2023. Para as análises, usamos os pressupostos da Teoria Clássica dos Testes, a taxonomia de Bloom e o coeficiente de fidedignidade alfa de Cronbach. Os itens se mostraram fáceis (índice de dificuldade média entre 0,3-0,4), com discriminação de regular a boa (índice de discriminação entre 0,3-0,4) e com predomínio de questões de média a alta taxonomia. A confiabilidade se manteve substancial ao longo dos anos (>0,6). O ganho de conhecimento dos estudantes em cirurgia é progressivo e mais importante a partir do 3º ano do curso de graduação, chegando a aproximadamente 70-75% no 6º ano. Este arcabouço de aferições pode ser replicado em outros contextos para melhor compreensão do aprendizado dos estudantes e para qualificação dos processos avaliativos.

## INTRODUCTION

The Progress Test (PT) is an assessment applied to medical students aiming to analyze the consecutive gain of knowledge throughout the course. The same test is applied to all students, from the first to the sixth year, with different tests for each application, which have a fixed periodicity and whose content is aimed at the level of the newly graduated doctor^1^.

PT was created in the Netherlands and the United States in the 1970s and aimed to change the culture of evaluating the teaching-learning process, with the principle of longitudinal evaluation and monitoring of the process effectiveness^2^ ^,^ ^3^. Today, PT is recognized for its potential to provide detailed feedback for students, teachers, and the medical school itself, providing information on personal, group, curriculum, and institution performance^4^. Furthermore, PT reduces the endogeneity effect of assessments conducted within the same school, as, working with consortia of schools, there are multiple sources of origin for items (questions)^5^. For this reason, PT has proven to be a useful predictor of performance in professional certification exams or for medical residency^6^ ^,^ ^7^.

In Brazil, PT has been used since the late 1990s and early 2000s^8^. With almost 20 years of experience, the Interinstitutional Center for Studies and Assessment Practices in Medical Education (NIEPAEM) is the consortium that brings together public medical schools in the State of São Paulo, and its practices have been the basis for replicating the model throughout Brazil^9^.

Traditionally, PT’s questions are divided into the areas stipulated for medical residency exams: clinics, pediatrics, surgery, gynecology, and public health^10^. This division has been questioned for giving equal weight to areas with heterogeneous content extension, which may compromise the reliability of the evidence in subareas and, therefore, of the evidence itself (unpublished data). Still, given the historical series of PT application, it would be possible to obtain information about the teaching of surgery in Brazil today, particularly in schools in São Paulo. Therefore, the objective of this study was to analyze the characteristics of the items and the performance of students in surgery PT from 2017 to 2023.

## METHODS

### Study design

This is a cross-sectional, observational, analytical study conducted using information from the Interinstitutional Center for Studies and Assessment Practices in Medical Education (NIPAEM) database. It is a consortium of the following medical schools: Paulista State University (UNESP), University of São Paulo (USP, courses in Ribeirão Preto and Bauru), State University of Campinas (UNICAMP), Federal University of São Paulo (UNIFESP), Federal University of São Carlos (UFSCar), State University of Londrina (UEL), Faculty of Medicine of São José do Rio Preto (FAMERP) and Faculty of Medicine of Marília (FAMEMA). Until 2022, the group had the participation of the Regional University of Blumenau (FURB). This is a study based on an aggregated database with individualized information at the item level (questions). Therefore, there is no individualized information on students (sex/age). As per the NIEPAEM code of conduct, there is also no identification of performance by institution, avoiding comparisons that lead to the classification of schools.

We included data from tests administered annually from 2017 onwards, including the first application of the test in 2023, when the test began to be administered bi-annually. For the psychometric data of the questions, we considered only the grades of students in the sixth year, as the test is formulated for the level of recent graduates. To analyze progress, we considered the performance of all students. Until 2022, the PT surgery section had 20 items per test. As of 2023, the section now has 23 items. Table 1 contains the matrix of question themes. This matrix is followed to prepare the test, to guarantee similarity of content in different applications.

**Table 1:** Knowledge matrix used to prepare the questions.

Subarea	Themes
Surgery general principles	Perioperative care
	Operative wounds
	Concepts of threads, sutures, and knots
General surgery	Appendicitis
	Hernias
Digestive system surgery	Esophageal, gastric, and colorectal lesions
	Liver, pancreas, and bile ducts
	Digestive bleeding/obesity
Pediatric surgery	Malformations of the gastrointestinal tract
	Testicular disorders/phimosis
Vascular surgery	Arterial and venous diseases
Thoracic surgery	Pleural neoplasms/infections/conditions
Urology	Nephrolithiasis
	Neoplasms of the genitourinary tract
Orthopedics	Fractures/joint injuries, musculoskeletal, ligaments/low back pain
Neurosurgery	Intracranial hypertension / traumatic brain injury / spinal cord trauma / malformations of the central nervous system
Plastic surgery	Burns/grafts and flaps
Head and neck surgery	Facial trauma/neoplastic lesions
Subarea	Themes
Ophthalmology	Eye trauma/red eye/reduced visual acuity
Anesthesiology	Types of anesthesia / pharmacology / pre-anesthetic assessment / anesthetic complications / pain
emergency medicine	Principles of ATLS, cardio-pulmonary resuscitation
	Thoracic, abdominal, pelvic, and vascular trauma

ATLS: Advanced Life Trauma Support.

For the analyses, we investigated the difficulty and discrimination index of the items (according to the Classical Test Theory), the taxonomic classification of the questions, and the test’s reliability coefficient (measured by Cronbach’s alpha coefficient).

### Ethical considerations

Because it deals with a database study made available in an aggregated form without the possibility of individual identification of students, this study does not need to be assessed by an Ethics in Research Committee, in accordance with the legislation of the National Commission for Ethics in Research with Human Beings (CONEP)^11^.

### Data analysis

The difficulty level of each item was calculated as the percentage of errors in each item (i.e., the closer to 1, the more difficult the question). To classify the degree of difficulty of each item, we adopted the following values: above 0.8 - difficult; between 0.4 and 0.8 - average; below 0.4 - easy.

We calculated the discrimination index by the difference in correct answers for each item between the 27% of students with higher performance on the test and the 27% with lower performance. Thus, the index can vary from -1 to 1, and the closer to 1, the better the discrimination. We adopted the following values to classify the questions: ≥0.4 - good; ≥0.3 and below 0.4 - regular; ≥0.2 and below 0.3 - weak; <0.2 - deficient.

We computed the alpha reliability coefficient for each test according to the formula proposed by Cronbach^12^. It refers to the internal consistency of the measure, that is, the extent to which the items measure the same construct. We adopted the following classification: > 0.8 -near perfect; from 0.8 to 0.61 - substantial; from 0.6 to 0.41 - moderate; from 0.4 to 0.21 - reasonable; <0.21 - small.

According to Bloom’s Taxonomy, later modified by Anderson and Krathwol, cognitive educational domains can be classified according to the complexity of cognitive processes into: knowledge, understanding, application, analysis, synthesis, and evaluation^13^ ^,^ ^14^. For taxonomic classification of items, they were classified according to the cognitive repertoire involved in their resolution as low (memorization), medium (understanding), or high taxonomy (application/analysis).

We calculated student performance as a function of the average percentage of correct answers for each year of graduation.

To analyze the temporal trend of psychometric indicators, we conducted a simple linear regression. In analyzing the difference in student performance, we performed a one-way ANOVA test followed by the Tukey test for paired comparisons between subsequent years of the undergraduate course. We considered p<0.05 as statistically significant.

The analyzes were conducted using GraphPad v. 9.5.0 (GraphPad Software Inc. San Diego, CA, USA) and SPSS (Statistical Package for Social Sciences, IBM Corp., Armonk, NY, USA).

## RESULTS

**Figure 1:**
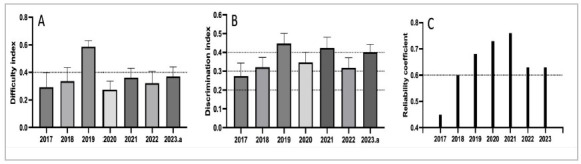
Mean values with respective 95% confidence intervals for the difficulty (A) and discrimination (B) indices of the surgery items for each application of the progress test in the years 2017 to 2023. C: coefficient reliability values of the surgery area for the same years.

Regarding the difficulty of the items, we observed that on the annual average, the items were easy, with an average difficulty index varying between 0.3 and 0.4 (Figure 1). Only the 2019 test had a higher average difficulty, close to 0.6. As for item discrimination, the average pointed to regular discrimination (between 0.3 and 0.4), with the 2019, 2021, and 2023 tests displaying an index close to or greater than 0.4 (good discrimination, Figure 1). The reliability analysis of the test, as measured by the reliability coefficient (Cronbach’s alpha), showed that, except for the 2017 test, all had a value greater than or equal to 0.6 (substantial internal consistency, Figure 1). In the temporal trend analysis of the indicators, all demonstrated stability: low angular coefficients and statistically non-significant (Table 2).. 

**Table 2:** Linear regression results for behavioral trends of psychometric indicators in surgery in the progress test from 2017 to 2023.

Indicator	Angular coefficient*	p-value
Difficulty Index	≅ 0.00%	0.978
Discrimination Index	1.24%	0.346
Reliability coefficient	2.43%	0.236

*High values of the angular coefficient indicate an increasing trend over time. Negative values indicate a downward trend. Values close to zero suggest stability.

**Figure 2:**
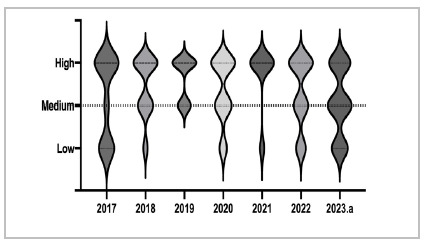
Violin plot for the number of items by taxonomic classification. The larger diameter of the violin indicates a greater concentration of items in that classification.

In the analysis of the items’ taxonomic classification, we observed a predominance of medium to high taxonomy questions (Figure 2), that is, there are few items that emphasize memorization of content and concepts, and more items that require greater cognitive complexity, with understanding, analysis, and application of contents.

**Figure 3:**
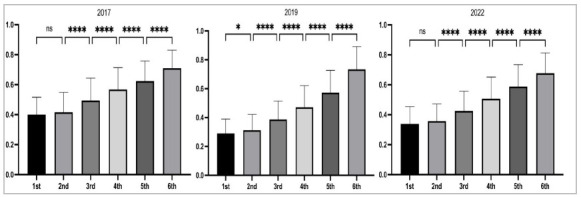
Performance of students in surgery in the progress test exemplified in the 2017, 2019, and 2022. The comparison of performance between subsequent series is always significant, except for the comparison between the first and second years. *p<0.05; ***p<0.0001; ns: not significant.

As for student performance, we observed that there were progressive gains with each year of graduation, starting from an average of 25 to 35% correct answers in the first year, reaching 70-75% in the sixth. When comparing subsequent graduation, performance was different for almost all years, except for the comparison between the first and second years, which showed no difference for the test applying in 2017, 2018, 2021, and 2022 (Figure 3).

## DISCUSSION

PT has been increasingly used in Brazilian medical schools. Faced with numerous discussions about the importance of external and serial assessments of medical students, PT appears to be a useful tool for allowing diagnoses on student performance and curriculum behavior and, ultimately, the effectiveness of the teaching-learning process^15^ ^,^ ^16^.

There are valid criticisms of regarding PT’s limitation for inferences about specific disciplines. For example, a student’s error on a single anatomy question does not mean that the student does not have the necessary cognitive skills about anatomy. Therefore, for more accurate analyzes of a particular area, more in-depth approaches to the test results are necessary, or an increase in sampling of the area in question by increasing the number of its items in the test^17^.

Therefore, we believe that this work, analyzing equivalent evidence in surgery (which makes up 1/6 to 1/5 of PT) across seven applications, keeping the matrix of covered knowledge fixed, allows for some conclusions.

Firstly, we noted that the surgery test is not a difficult one. The questions have low average difficulty ratings (below 0.4) and have remained stable over the years. Knowing that the contents covered in the test are at the level of a newly qualified doctor, this is a good indicator.

This observation is reinforced by the average discrimination index, which has also remained stable and above 0.3, therefore, with items regulating good discrimination. The interpretation of this indicator is that the items have been adequate in detecting students with good or deficient performance. Therefore, even on easy questions, schools and mentors can identify students with unsatisfactory performance, who deserve attention. We should emphasize that the 2019, 2021, and 2022 tests used pre-tested items, that is, they were items already used in previous PT versions and were chosen based on their good psychometric behavior. It is not by chance, therefore, that these were the tests in which we observed the best item discrimination averages.

This observation has an important practical implication for medical schools. Teachers usually repeat test questions - either due to lack of organization of a proper database, or due to limited creativity in writing new items^18^ ^-^ ^20^. It is recommended that items be reused with sufficient time intervals to minimize biases in remembering questions among students and their peers. With our data, we draw attention to the fact that teachers, in addition to ensuring intervals in the application of repeated items, should study the psychometric behavior of the questions after their use, identifying questions that are very easy, very difficult, or that do not show good discrimination, that is, do not contribute to an adequate assessment^21^. The psychometric analysis of items can be done automatically on online assessment platforms, the use of which gained notoriety with the COVID-19 pandemic^22^.

The reliability coefficient is also an indicator that the PT surgery test is of superior quality, with indices that have remained above 0.6 and, therefore, with substantial internal consistency and comparable to values obtained internationally^23^. Obviously, the set of all items that comprise the test raises the coefficient to values above 0.8-0.9 (unpublished data). It must be recognized, however, that this coefficient is influenced by the score variance and, therefore, by the number of respondents^21^. As we have students from nine schools, the sample size is large and naturally increases the variance of responses and the value of Cronbach’s alpha. However, the set of different psychometric indicators taken together suggests that the assessment has had a satisfactory quality.

To this set of indicators, we added the items’ taxonomic category, with a predominance of medium to high taxonomy questions. It has been demonstrated that items with a higher taxonomy have a better discrimination index than items with a lower one^24^ ^,^ ^25^. Thus, the PT surgery items have required more clinical reasoning from students than memorization of facts or concepts, possibly approaching cognitive domains closer to the clinical practice of the newly graduated doctor.

Finally, regarding the performance of medical students, we observed gains in knowledge throughout the course, as expected. The interesting fact is that the gain is significant from the third year onwards, which reflects the reality of the curriculum of most Brazilian medical schools, in which the teaching of surgical technique and clinical practice occurs more frequently in the third year. Recently, it was demonstrated that curricular exposure of students to surgery content improves their performance on PT surgery items, although performance at the end of the course is similar among students, regardless of the curricular design^26^. The fact that sixth-year students have an average success rate close to 70-75% is comparable to that of other areas of knowledge and to what is reported in the international literature on PT^2^ ^,^ ^27^ ^,^ ^28^.

This study is not without limitations. Due to the very nature of PT, we cannot infer conclusions about specific learning in each surgical specialty. Obviously, as this is a knowledge assessment, it is also not possible to make any inferences about the teaching and learning of basic surgical skills that every doctor should have, nor about professional attitudes. It should also be noted that the data from the 2023 test correspond to the test administered at the end of the first semester, and not at the end of the year, since this year the frequency of the test was increased to twice a year. Furthermore, we do not have information at the individual student level to make other inferences based on covariates such as sex, age, and institution. As per the NIEPAEM code of conduct, each student’s performance information is only available to the student’s own school, and not to the group of schools.

Despite these limitations, this study provides useful information about the quality of PT for assessing surgical knowledge and provides more evidence about the knowledge gain curve of medical students. Together, we present a framework of assessment quality measurements that can be repeated in other contexts to qualify medical student assessment.

## CONCLUSION

The surgery items that comprise the NIEPAEM Progress Test are not difficult, have good discrimination, favor clinical reasoning, and produce good reliability indicators. Students’ knowledge gain is significant from the third year of the undergraduate course and reaches 70 75% by the sixth year.
